# Intracoronary Adenosine–Derived Coronary Flow Reserve and Index of Microvascular Resistance

**DOI:** 10.1161/CIRCINTERVENTIONS.123.013667

**Published:** 2024-05-08

**Authors:** Daniel T.Y. Ang, David Carrick, Anna Kamdar, Robert Sykes, Ross J. McGeoch, Damien Collison, Alex McConnachie, Colin Berry

**Affiliations:** West of Scotland Regional Heart and Lung Centre, Golden Jubilee National Hospital, Clydebank, United Kingdom (D.T.Y.A., A.K., R.S., D. Collison, C.B.).; British Heart Foundation, Glasgow Cardiovascular Research Centre, School of Cardiovascular and Metabolic Health (D.T.Y.A., A.K., R.S., C.B.), University of Glasgow, United Kingdom.; Robertson Centre for Biostatistics, School of Health and Wellbeing (A.M.), University of Glasgow, United Kingdom.; Department of Cardiology, University Hospital Hairmyres, Lanarkshire, United Kingdom (D.T.Y.A., D. Carrick, R.J.M.).

**Keywords:** coronary angiography, coronary artery disease, hyperemia, infusions, intravenous, microvascular angina

Coronary microvascular dysfunction causes myocardial ischemia with no obstructive coronary artery disease,^1^ angina, and impaired quality of life.^2^ Coronary function testing undertaken during invasive coronary angiography may aid diagnosis.^3^ Intravenous adenosine infusion for the induction of stable hyperemia is the reference approach for assessing microvascular function.^3^ However, systemic side effects, logistics, and cost limit clinical adoption.^4^ Intracoronary adenosine (low-dose defined as <100 µg) represents an alternative method for achieving myocardial hyperemia, with fewer side effects and improvements in logistics.^4,5^ Furthermore, the onset of hyperemia is faster (<5 seconds) after intracoronary adenosine compared with intravenous infusion (1–2 minutes).^4,5^ Accordingly, we investigated the feasibility and diagnostic value of intracoronary adenosine–derived indices of microvascular function.

We performed coronary function testing with a thermistor/pressure sensor diagnostic guidewire (PressureWire X, Abbott Vascular) in 100 coronary arteries from 76 consecutive patients undergoing clinically indicated invasive coronary angiography for the investigation of suspected angina. The study was undertaken between November 2022 and September 2023 in 2 regional cardiac centers and received approval by local institutional review bodies. The sample size was prespecified to include 100 prospective evaluations. All participants provided written informed consent. The data that support the findings of this study are available from the corresponding author upon reasonable request.

With the diagnostic guidewire positioned in the distal (>6 cm from ostium) coronary artery, resting thermodilution bolus injections of 3 mL of room-temperature normal saline were performed. Coronary physiology responses were measured using linked software (CoroFlow, v3.01, Coroventis). Next, a low-dose (90-µg) intracoronary rapid bolus of adenosine was administered through the guiding catheter, which was then quickly purged with room-temperature normal saline. Three coronary thermodilution measurements were performed in rapid succession during the resultant hyperemic phase. Following a 2-minute rest period, the hyperemic transit time protocol was repeated using high-dose (210-µg) intracoronary adenosine. Finally, intravenous adenosine infusion (140 µg/kg per min) was commenced, and coronary thermodilution was repeated during systemic hyperemia.

Coronary flow reserve (CFR) and index of microvascular resistance (IMR) were calculated using mean (MeanTmn), first (1stTmn), and minimum (MinTmn) transit times for low- and high-dose intracoronary adenosines. Receiver operating characteristic (ROC) curves were used to determine the accuracy of each test (area under the ROC curve) and identify optimal cutoffs corresponding to intravenous adenosine CFR <2.0 and IMR ≥25 (Youden index).

The study population (n=76) included 33 (43.4%) women, with a median age of 62 (interquartile range, 57–68) years. The clinical presentation was stable angina in 90.8% of patients and non-ST elevation acute coronary syndromes in the remainder. Medical history included smoking history in 52.6%, hypertension in 52.6%, diabetes in 14.5%, chronic kidney disease in 2.6%, atrial fibrillation in 9.2%, previous myocardial infarction in 27.6%, and stroke in 2.6%.

Of the 100 coronary arteries evaluated, 66 coronary function tests involved the left anterior descending, left circumflex in 28, and right coronary artery in 6. The median (interquartile range) left ventricular end-diastolic pressure was 7 (5–10) mm Hg, intravenous adenosine fractional flow reserve was 0.89 (0.83–0.94), intravenous adenosine CFR was 3.1 (2.1–4.7), and IMR was 17 (13–28). Generally, transit times after intracoronary adenosine demonstrated sequential prolongation, illustrating the offset of hyperemia. On ROC analysis, low-dose intracoronary adenosine demonstrated greater accuracy versus high-dose intracoronary adenosine (Figure) for predicting intravenous adenosine–derived CFR and IMR. Specifically, indices derived from MeanTmn values outperformed those calculated from 1stTmn and MinTmn. Low-dose intracoronary adenosine CFR (area under the ROC curve, 0.97 [95% CI, 0.94–1.00]) cutoff of <2.8 had the best sensitivity (100.0%), specificity (81.6%), positive predictive value (63.2%), and negative predictive value (100.0%) by the Youden index for predicting intravenous adenosine CFR <2.0. Low-dose intracoronary adenosine IMR (area under the ROC curve, 0.93 [95% CI, 0.88–0.98]) cutoff of ≥20 had the best sensitivity (96.8%), specificity (78.3%), positive predictive value (66.7%), and negative predictive value (98.2%) by the Youden index for predicting intravenous adenosine IMR ≥25. For high-dose intracoronary adenosine, CFR <2.5 and IMR ≥20 performed the best in predicting abnormal intravenous indices. There were good correlations between MeanTmn-derived low-dose intracoronary adenosine CFR and intravenous adenosine CFR (r=0.79; r^2^=0.62; and *P*<0.001) and MeanTmn-derived low-dose intracoronary adenosine IMR and intravenous adenosine IMR (r=0.78; r^2^=0.60; and *P*<0.001). Bland-Altman plots show no evidence of bias overall or in relation to the mean (95% limits of agreement ≈±0.3 on a log scale).

**Figure.**
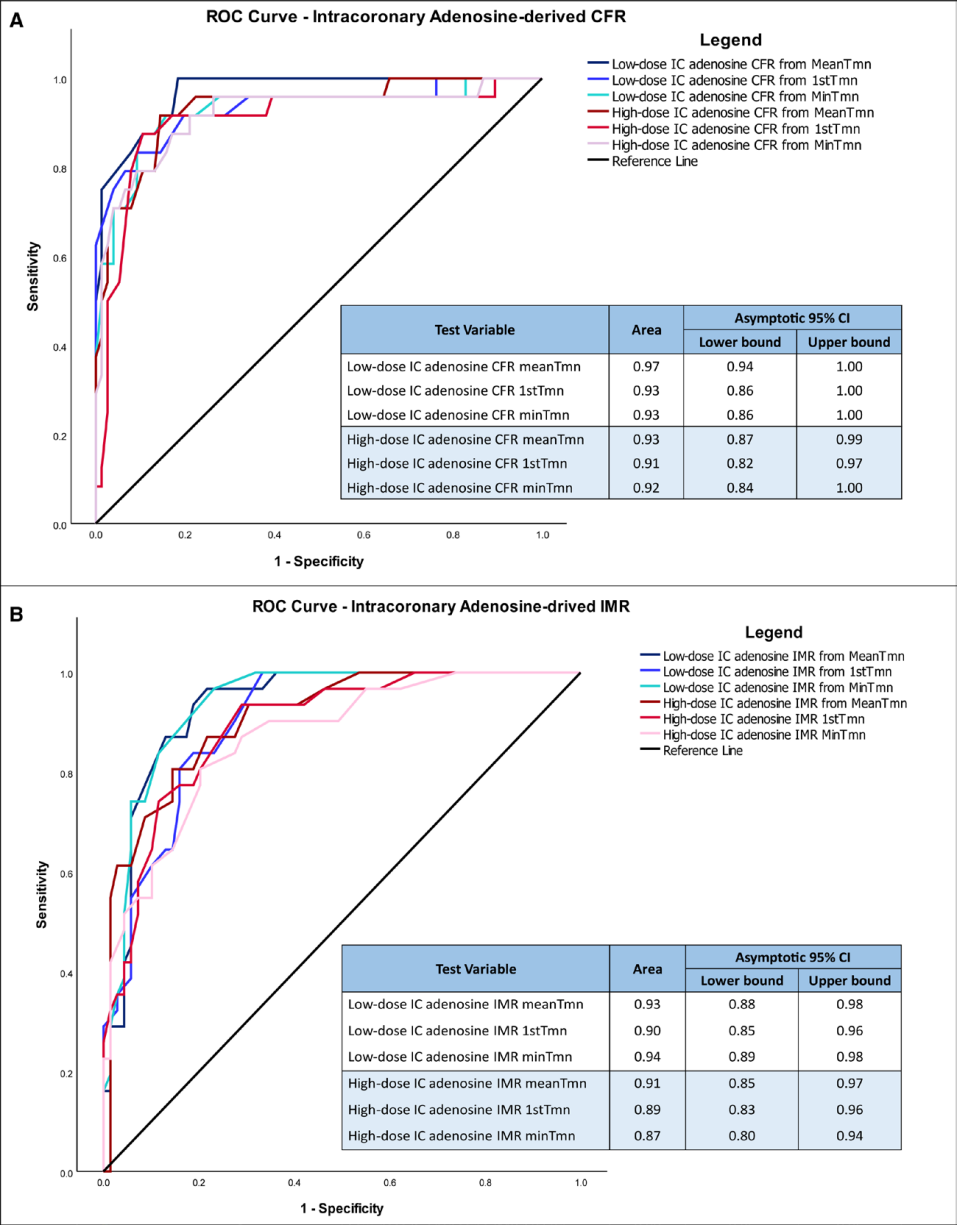
**Summary of the performance of intracoronary (IC) adenosine-derived indices of coronary microvascular function.** Receiver operating characteristic (ROC) curves comparing the accuracy of low-dose (90 µg) vs high-dose (210 µg) IC adenosine–derived coronary flow reserve (CFR; **A**) and index of microvascular resistance (IMR; **B**). CFR and IMR derived from the mean transit times (indices derived from the mean value of all 3 thermodilution transit times [MeanTmn]) after low-dose IC adenosine were best at predicting intravenous adenosine–derived CFR <2.0 and IMR ≥25. 1stTmn indicates thermodilution transit time from the first injection in the sequence; and MinTmn, minimum thermodilution transit time.

Regarding safety, transient (<5 seconds, self-limiting) atrioventricular conduction block occurred in one patient with low-dose intracoronary adenosine and 17 patients with high-dose intracoronary adenosine. Four patients (5.3%) reported transient chest discomfort or dyspnea during intracoronary adenosine administration versus 72 (94.7%) with intravenous adenosine (McNemar *P*<0.001).

Study limitations include the transient hyperemic effect of intracoronary adenosine necessitating meticulous guiding catheter management to selectively deliver intracoronary adenosine followed by catheter purging with saline before recording hyperemic thermodilution response.

Our study provides novel data on the feasibility and diagnostic value of intracoronary adenosine for estimating CFR and IMR compared with systemic hyperemia using intravenous adenosine. A hybrid algorithm incorporating intracoronary adenosine CFR ≥2.8 and IMR <20 for the rapid exclusion of abnormal intravenous CFR/IMR may encourage the uptake of microvascular function testing among clinicians. External validation in larger populations is warranted.

## ARTICLE INFORMATION

### Acknowledgments

The authors thank the patients and staff who supported this project.

### Sources of Funding

This research was supported by the British Heart Foundation (grant RE/18/6/34217) and Abbott Vascular. Dr Berry receives research funding from the British Heart Foundation (grants RE/18/6/34217 and PG/19/28/34310), the Chief Scientist Office, Engineering and Physical Sciences Research Council (grants EP/R511705/1 and EP/S030875/1), and the Medical Research Council (grant MR/S018905/1).

### Disclosures

Dr Berry is employed by the University of Glasgow that holds consultancy and research agreements for his work with Abbott Vascular, AstraZeneca, Boehringer Ingelheim, Coroventis, HeartFlow, Menarini, MSD, Novartis, Servier, Siemens Healthcare, TherOx, Inc, and Valo Health. The other authors report no conflicts.
